# Biomarker implication of kallikrein-related peptidases as prognostic tissue substrates of poor survival in colorectal cancer

**DOI:** 10.1186/s12935-020-01350-4

**Published:** 2020-06-22

**Authors:** Qiliang Peng, Yi Shen, Peifeng Zhao, Ming Cheng, Yongyou Wu, Yaqun Zhu

**Affiliations:** 1 Department of Radiotherapy & Oncology, The Second Affiliated Hospital of Soochow University, San Xiang Road No. 1055, Suzhou, 215004 Jiangsu China; 2 Institute of Radiotherapy & Oncology, Soochow University, Suzhou, China; 3 Department of Radiation Oncology, The Affiliated Suzhou Science & Technology Town Hospital of Nanjing Medical University, Suzhou, China; 4 Department of General Surgery, The Second Affiliated Hospital of Soochow University, Suzhou, China

**Keywords:** Colorectal cancer, Kallikrein, Prognosis, Biomarker

## Abstract

**Background:**

Recent studies have demonstrated that the kallikrein and kallikrein-related peptidases (KLKs) exhibit aberrant expression in patients with colorectal cancer (CRC) and might be considered as potential prognostic biomarkers of CRC. However, inconsistent findings have been reported, which promote us to summarize the global prognostic roles of KLKs for survival in CRC patients.

**Methods:**

Eligible published studies were identified by searching electronic databases with several search strategies. The patients’ baseline characteristics and survival results were extracted from enrolled studies and pooled as combined hazard ratio (HR) with 95% confidence interval (95% CI) to estimate the effect size.

**Results:**

A total of 25 and 22 eligible studies were included in the meta-analysis to evaluate the prognostic roles of KLKs on overall survival (OS) and disease-free survival (DFS), respectively. KLKs overexpression was significantly associated with worse OS (pooled HR = 1.43, 95% CI 1.27–1.60, P < 0.001) and short DFS (pooled HR = 1.35, 95% CI 1.21–1.51, P < 0.001). Importantly, subgroup and meta-regression analyses revealed the survival differences among different races and detection methods of KLKs. Furthermore, several specific members of KLKs were identified to be more significantly related to worse OS and DFS compared with other members.

**Conclusion:**

The present study demonstrated that KLKs may have the potential to serve as promising biomarkers to monitor CRC prognosis and progression. The promising results concerning the utility of KLKs in clinical practice encourage the further investigation of their clinical utility applicability as tumor markers of CRC.

## Background

According to recent cancer statistics, colorectal cancer (CRC) remains as one of the most commonly diagnosed malignancy and one of the leading causes of death from cancer worldwide [1]. Despite increased early detection of CRC in recent years and improved survival benefit provided by curative surgery and adjuvant chemotherapy/radiotherapy, the prognoses of these patients are still poor and unsatisfied due to the high recurrence rates and distant metastases [2]. Tumor biomarkers are helpful to refine prognostication and predict the benefit derived from systemic treatment of CRC patients. However, the use of serum-based tumor biomarkers has a limited role due to lack of specificity and sensitivity [3]. As a consequence, it is necessary to explore novel and suitable biomarkers to predict the survival and provide information for clinical treatment.

Serine proteases are a subgroup of enzymes that utilize a uniquely activated serine residue to catalytically hydrolyze peptide bonds, playing important and vital role in cell growth regulation, invasion, and angiogenesis [4]. Among all serine proteases within the human genome, the kallikrein and kallikrein-related peptidases (KLKs) clusters are the largest [5]. The family of tissue KLKs consists of 15 secreted serine proteases encoded by conserved genes (KLK1–KLK15), which are localized in tandem on chromosomal region 19q13.4. Accumulating evidence has indicated that many members of the human tissue KLKs are differentially expressed in many pathological conditions and a number of malignancies and may have clinical utility as cancer diagnostic/prognostic biomarkers [6]. Since then, the prognostic significance of several KLKs in CRC has been intensively investigated. Protein expression analysis of a panel of KLKs in cytosolic extracts from CRC tissues revealed that KLKs are aberrantly expressed in colorectal tumors, compared with their noncancerous counterparts [7]. Given their critical involvement in the vital biological processes and unique biomarker features mentioned above, KLKs could be considered as good candidates in increasing the accuracy of prediction of patients’ survival beyond the traditional clinical information.

Although some of the studies evaluated the prognostic value of KLKs in CRC patients, the relationships between KLKs and CRC remain controversial as individual studies were not comprehensive for they involved only small study populations. For instance, Christodoulou et al. [8] showed that increased level of KLK6 was associated with worse overall survival of CRC patients; nevertheless, this association has not been detected in the study illustrated by Vakrakou et al. [9]. Therefore, in the present study, we conducted this comprehensive meta-analysis to compare the survival outcome between CRC patients with high levels of KLKs and those with low levels of KLKs. We aimed to overcome the limitation of the single study and to obtain a better understanding of the prognostic value of KLKs in CRC.

## Materials and methods

### Publication search

The present study was conducted and reported under the guidelines formulated in Preferred Reporting Items for Systematic Reviews and Meta-analyses (PRISMA). A comprehensive literature search was carried out based on the electronic databases including PubMed, EMBASE, Cochrane Library, and Web of Science databases (up to March 12, 2019) by using the following keywords: (“Kallikrein”OR “KLK” OR “Kallikrein-related peptidase”), (“rectal” OR “rectum” OR “colon” OR “colorectal” OR “CRC”), and (“cancer” OR “tumor” OR “neoplasm” OR “carcinoma”). All potentially eligible studies were identified and their bibliographies were carefully examined to retrieve other eligible studies.

In case of omission, additional relevant studies were identified by scanning the references cited in the original studies. Two reviewers (Peng and Shen) independently performed the publication search and the following steps.

### Inclusion criteria

The studies qualified to be included had to meet the following criteria: (1) they investigated the relationships between KLKs expression and CRC prognosis; (2) they reported survival data for OS or DFS; (3) they directly provided HRs with 95% CIs or providing adequate statistics to conjecture HRs with a corresponding 95% CI.

### Exclusion criteria

The studies were excluded if (1) they were not pertinent to KLKs; (2) they published as reviews, letters, case reports, editorials, or expert opinions; (3) they were non-English publications; or (4) they lacked sufficient data for further quantification.

### Data extraction

According to the inclusion and exclusion criteria above, data were collected carefully and independently by two researchers (Peng and Shen) from all eligible publications based on standardized forms. Any disagreement between the researchers was resolved by consulting with a third investigator (Zhao) through independently extracting data from the enrolled publication and then reaching a consensus by discussions. The following characteristics from each study were extracted: first author, publication year, study population, patient characteristics (age, gender, cancer type, etc.), number of patients, methods of KLKs detection, prognostic results including follow-up time and HRs estimates with 95% CIs for DFS or OS. If the survival data (HRs and 95% CIs) were not directly reported in the original study, they were obtained from Kaplan–Meier survival curves with the Engauge Digitizer V4.1 and estimated using the method previously introduced by Tierney et al. [10]. We also contacted the authors of eligible studies by email for additional information and the essential data required for the meta-analytic calculations.

### Quality assessment

The methodological quality assessment of each eligible article was conducted following the guidelines of the Newcastle–Ottawa Scale (NOS), which assessed studies with 9 items including the selection of the patient population, study comparability, outcome of interest, follow-up et al. [11]. Studies with an NOS score ranging from 6 to 8 were considered of high quality.

### Statistical analysis

In order to assess the associations between KLKs expression and the survival of CRC patients, pooled HRs with the corresponding 95% CIs were used to assess the strength of the associations between KLKs and the clinical prognosis of CRC patients. Heterogeneity across studies was checked using Cochran’s Q test (significant at P < 0.05) and Higgins’s I^2^ statistic (ranging from 0 to 100%) [12]. For the presence of heterogeneity (P < 0.05, I^2^ > 50%), a random-effect model was employed to calculate the pooled HRs and 95% CIs; otherwise, a fixed effect model was selected (P > 0.05, I^2^ < 50%). Potential sources of heterogeneity were explored by performing meta-regression, subgroup, and sensitivity analyses [13]. At last, Begg’s funnel plots and Egger’s test were utilized to assess the included studies for the possible publication bias [14]. The statistical analyses were carried out using STATA (version 14.0) statistical software. Values of P < 0.05 were considered as statistical significance, except those for heterogeneity.

## Results

### Demographic characteristics

**Fig. 1:**
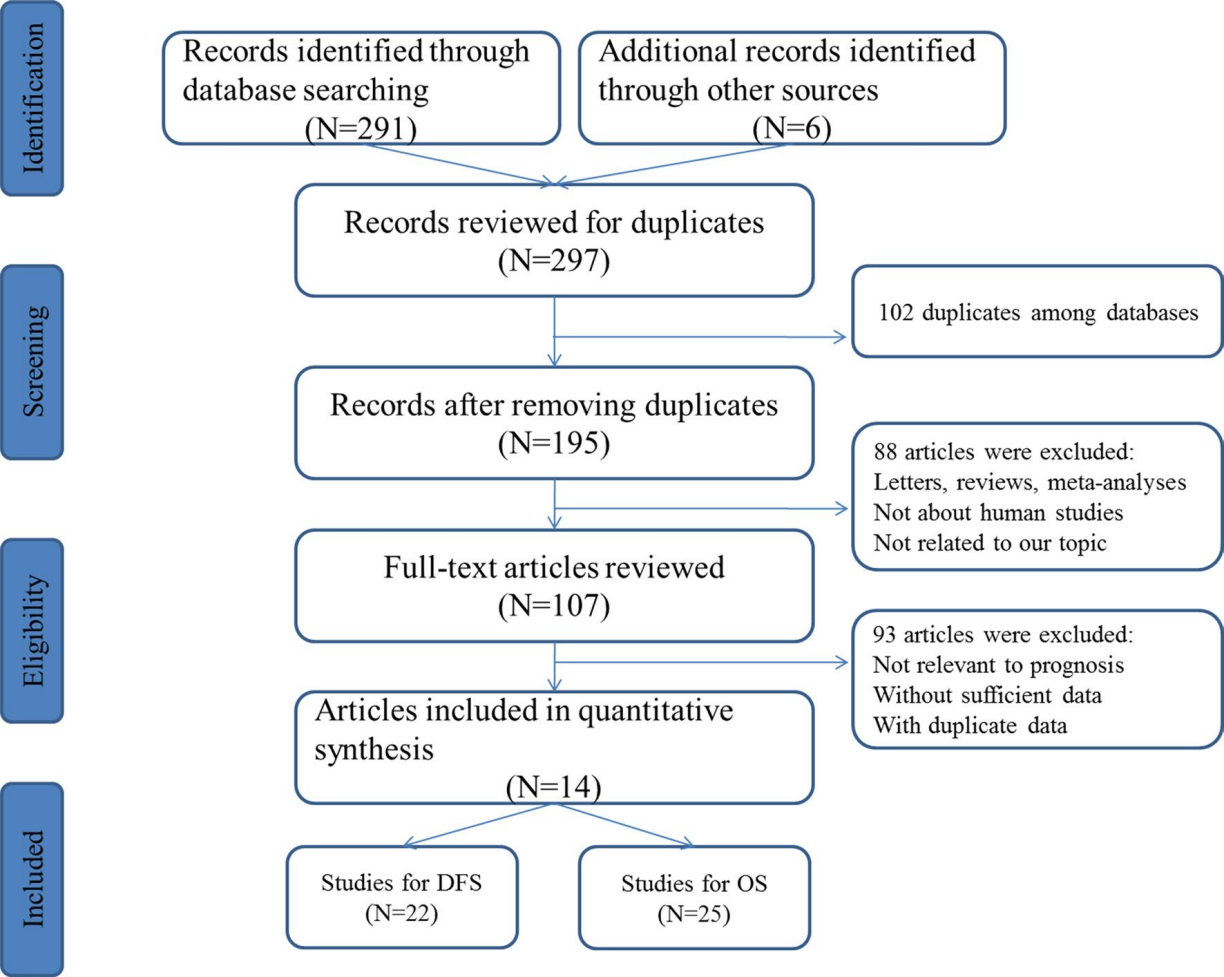
Flow diagram of the study selection process

Using different combinations of key terms, the initial search from the selected literature databases (PubMed, EMBASE, Cochrane Library, and Web of Science databases) and other sources retrieved a total of 297 records. As shown in the flow diagram for the literature (Fig. 1), after careful exclusion of inappropriate ones in each step, 14 articles including 25 studies for OS and 22 studies for DFS that met the inclusion norm were finally enrolled for the evidence synthesis, which evaluated the relevance between KLKs expression and CRC prognosis [8, 9, 15–25]. The main features of all the eligible studies are summarized in Tables 1 and 2. Among all cohorts, Caucasian (20 studies both for OS and DFS) became the major race of literatures, followed by Asian (5 for OS and 2 for DFS). Three methods were applied to measure the expression including quantitative real-time polymerase chain reaction (qRT-PCR), immunohistochemistry (IHC) and enzyme-linked immunosorbent assay (ELISA). Several members of KLKs were evaluated by more studies (n >=3) including KLK6 (n = 6), KLK7 (n = 4), KLK10 (n = 4), KLK11 (n = 3) for OS and KLK6 (n = 5), KLK7 (n = 3), KLK10 (n = 4) for DFS.

**Table 1:** The main features of enrolled studies for evaluating DFS

First author	Year	Ethnicity	KLK type	Sample	Sample size	Detection method	Mean age	TNM stage	Median follow-up time	Hazard ratio
Talieri et al.	2009	Caucasian	KLK7	Tissue	98	qRT-PCR	67.4	I–IV	29	2.05 (1.05–1.00)
Talieri et al.	2009	Caucasian	KLK5	Tissue	127	ELISA	68.7	I–IV	37.2	1.16 (0.99–1.37)
Talieri et al.	2009	Caucasian	KLK6	Tissue	128	ELISA	68.7	I–IV	37.2	1.09 (0.90–1.33)
Talieri et al.	2009	Caucasian	KLK7	Tissue	128	ELISA	68.7	I–IV	37.2	1.28 (0.96–1.72)
Talieri et al.	2009	Caucasian	KLK8	Tissue	128	ELISA	68.7	I–IV	37.2	1.00 (0.80–1.26)
Talieri et al.	2009	Caucasian	KLK10	Tissue	128	ELISA	68.7	I–IV	37.2	1.07 (0.92–1.23)
Talieri et al.	2009	Caucasian	KLK11	Tissue	127	ELISA	68.7	I–IV	37.2	1.12 (0.90–1.39)
Talieri et al.	2009	Caucasian	KLK13	Tissue	128	ELISA	68.7	I–IV	37.2	1.29 (0.98–1.71)
Talieri et al.	2009	Caucasian	KLK14	Tissue	128	ELISA	68.7	I–IV	37.2	1.33 (1.05–1.68)
Talieri et al.	2009	Caucasian	KLK15	Tissue	128	ELISA	68.7	I–IV	37.2	1.12 (0.85–1.46)
Talieri et al.	2011	Caucasian	KLK10	Tissue	119	qRT-PCR	67.45	I–IV	29	2.46 (1.01–6.09)
Kim et al.	2011	Asian	KLK6	Tissue	143	IHC	NA	I–IV	NA	1.98 (1.18–3.31)
Petraki et al.	2012	Caucasian	KLK6	Tissue	56	IHC	71	I–III	62	1.31 (0.94–1.81)
Petraki et al.	2012	Caucasian	KLK10	Tissue	56	IHC	71	I–III	62	2.54 (0.97–6.64)
Alexopoulou et al.	2013	Caucasian	KLK10	Tissue	121	qRT-PCR	66.5	NA	48.8	2.36 (1.09–5.08)
Kontos et al.	2013	Caucasian	KLK4	Tissue	62	qRT-PCR	66.7	I–IV	NA	2.73 (1.04–7.13)
Devetzi et al.	2013	Caucasian	KLK7	Tissue	95	qRT-PCR	68.4	I–IV	NA	1.47 (0.82–2.64)
Devetzi et al.	2013	Caucasian	KLK14	Tissue	95	qRT-PCR	68.4	I–IV	NA	1.80 (1.10–2.93)
Vakrakou et al.	2014	Caucasian	KLK6	Tissue	92	qRT-PCR	68.2	I–IV	NA	2.85 (0.98–8.27)
Christodoulou et al.	2014	Caucasian	KLK6	Tissue	110	qRT-PCR	65.5	I–IV	NA	4.47 (1.58–12.64)
Alexopoulou et al.	2014	Caucasian	KLK11	Tissue	120	qRT-PCR	69	I–IV	NA	2.89 (1.01–8.30)
Liu et al.	2017	Asian	KLK8	Tissue	124	IHC	NA	I–IV	NA	2.96 (1.48–5.94)

*DFS* disease-free survival, *qRT-PCR* quantitative real-time polymerase chain reaction, *IHC* immunohistochemistry, *ELISA* enzyme-linked immunosorbent assay, *NA* not available

**Table 2:** The main features of enrolled studies for evaluating OS

First author	Year	Ethnicity	KLK type	Sample	Sample size	Detection method	Mean age	TNM stage	Median follow-up time	Hazard ratio
Ogawa et al.	2005	Asian	KLK6	Tissue	63	qRT-PCR	NA	I–IV	30	1.35 (1.13–1.61)
Talieri et al.	2009	Caucasian	KLK7	Tissue	98	qRT-PCR	67.4	I–IV	29	2.87 (1.33–6.19)
Talieri et al.	2009	Caucasian	KLK5	Tissue	126	ELISA	68.7	I–IV	37.2	1.24 (1.05–1.47)
Talieri et al.	2009	Caucasian	KLK6	Tissue	127	ELISA	68.7	I–IV	37.2	1.09 (0.85–1.39)
Talieri et al.	2009	Caucasian	KLK7	Tissue	127	ELISA	68.7	I–IV	37.2	1.57 (1.04–2.37)
Talieri et al.	2009	Caucasian	KLK8	Tissue	127	ELISA	68.7	I–IV	37.2	1.01 (0.78–1.32)
Talieri et al.	2009	Caucasian	KLK10	Tissue	127	ELISA	68.7	I–IV	37.2	1.12 (0.94–1.34)
Talieri et al.	2009	Caucasian	KLK11	Tissue	126	ELISA	68.7	I–IV	37.2	1.16 (0.91–1.49)
Talieri et al.	2009	Caucasian	KLK13	Tissue	127	ELISA	68.7	I–IV	37.2	1.36 (1.00–1.87)
Talieri et al.	2009	Caucasian	KLK14	Tissue	127	ELISA	68.7	I–IV	37.2	1.43 (1.05–1.94)
Talieri et al.	2009	Caucasian	KLK15	Tissue	127	ELISA	68.7	I–IV	37.2	1.02 (0.77–1.36)
Yu et al.	2010	Asian	KLK11	Tissue	126	IHC	59.2	I–IV	NA	1.22 (0.71–2.32)
Inoue et al.	2010	Asian	KLK7	Tissue	136	qRT-PCR	NA	I–IV	NA	2.97 (1.36–6.30)
Talieri et al.	2011	Caucasian	KLK10	Tissue	119	qRT-PCR	67.4	I–IV	29	1.78 (0.67–4.74)
Kim et al.	2011	Asian	KLK6	Tissue	143	IHC	NA	I–IV	NA	2.22 (1.24–3.98)
Petraki et al.	2012	Caucasian	KLK6	Tissue	56	IHC	71	I–III	62	1.42 (1.02–2.01)
Petraki et al.	2012	Caucasian	KLK10	Tissue	56	IHC	71	I–III	62	3.63 (1.37–9.63)
Alexopoulou et al.	2013	Caucasian	KLK10	Tissue	121	qRT-PCR	66.5	NA	48.8	2.75 (1.16–6.52)
Kontos et al.	2013	Caucasian	KLK4	Tissue	62	qRT-PCR	66.7	I–IV	NA	1.07 (0.44–2.57)
Devetzi et al.	2013	Caucasian	KLK7	Tissue	95	qRT-PCR	68.4	I–IV	NA	1.93 (1.06–3.54)
Devetzi et al.	2013	Caucasian	KLK14	Tissue	95	qRT-PCR	68.4	I–IV	NA	2.18 (1.32–3.61)
Vakrakou et al.	2014	Caucasian	KLK6	Tissue	92	qRT-PCR	68.2	I–IV	NA	2.71 (0.91–8.08)
Christodoulou et al.	2014	Caucasian	KLK6	Tissue	110	qRT-PCR	65.5	I–IV	NA	3.65 (1.27–10.46)
Alexopoulou et al.	2014	Caucasian	KLK11	Tissue	120	qRT-PCR	69	I–IV	NA	4.06 (1.18–13.94)
Liu et al.	2017	Asian	KLK8	Tissue	124	IHC	NA	I–IV	NA	2.07 (1.11–3.87)

*OS* overall survival, *qRT-PCR* quantitative real-time polymerase chain reaction, *IHC* immunohistochemistry, *ELISA* enzyme-linked immunosorbent assay, *NA* not available

The Newcastle–Ottawa Scale (NOS) score of each study included for the evaluation of OS and DFS ranged from 7 to 9, which indicated that the quality of the included studies was moderate to high.

### Impact of KLKs expression on DFS

**Fig. 2:**
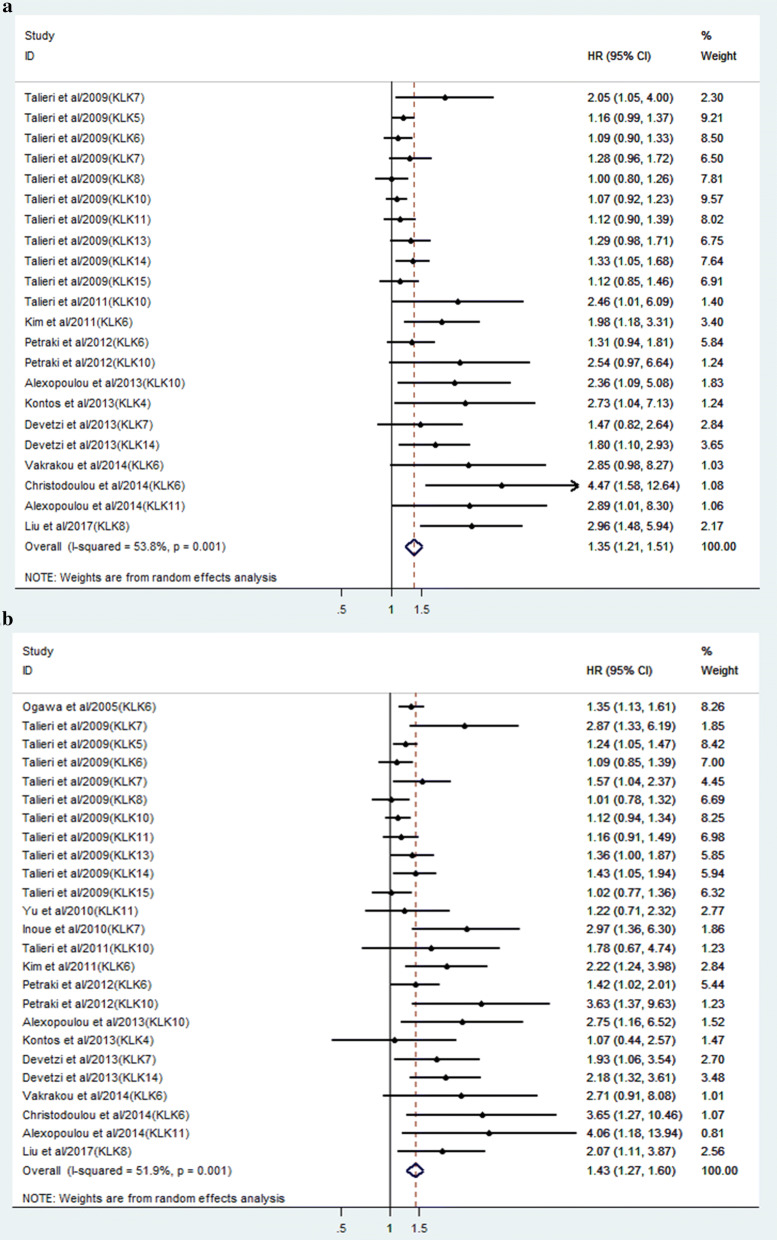
Forest plots of the correlation between KLKs expression level and CRC prognosis. **a** Forest plot of DFS; **b** forest plot of OS

A random-effects model was applied to estimate the pooled HR and corresponding 95% CI as the heterogeneity test reported the P value of 0.001 and I^2^ values of 53.8%. The pooled result revealed that elevated KLKs expression was significantly associated with worse DFS of patients with CRC (pooled HR = 1.35, 95% CI = 1.21–1.51, P < 0.001) (Fig. 2).

To explore the sources of heterogeneity, subgroup analysis was performed (Table 3). The results suggested that the associations between KLKs overexpression and worse DFS were significant in Caucasian patients (pooled HR = 1.28, 95% CI 1.16–1.43, P < 0.001), and more pronounced in Asian (pooled HR = 2.28, 95% CI 1.51–3.46, P < 0.001). For the analysis stratified by KLKs member, significant worse DFS was observed in KLK6 (HR = 1.63, 95% CI 1.12–2.39, P = 0.012), KLK7 (HR = 1.39, 95% CI 1.09–1.78, P = 0.007) and KLK10 (HR = 1.79, 95% CI 1.00–3.20, P = 0.05). Interestingly, when it came to the subgroup analysis by detection methods of KLKs, the results suggested the associations between poor DFS and KLKs overexpression were detected by all the three methods (qRT-PCR: pooled HR = 2.12, 95% CI 1.66–2.73, P < 0.001; IHC: pooled HR = 1.89, 95% CI 1.27–2.81, P = 0.002; ELISA: pooled HR = 1.13, 95% CI 1.06–2.81, P < 0.001).

**Table 3:** Results of subgroup analysis of studies for evaluating DFS

Subgroup	Number of studies	HR (95% CI)	P_HR_	Heterogeneity (I^2^)	P_heterogeneity_
Ethnicity					
Caucasian	20	1.28 (1.16–1.43)	< 0.001	46.5%	0.001
Asian	2	2.28 (1.51–3.46)	< 0.001	0%	0.012
KLK member					
KLK4	1	2.73 (1.04–7.15)	–	–	–
KLK5	1	1.16 (0.99–1.37)	–	–	–
KLK6	5	1.63 (1.12–2.39)	0.012	69.4%	0.011
KLK7	3	1.39 (1.09–1.78)	0.007	0%	0.441
KLK8	2	1.63 (0.57–4.71)	0.363	88.2%	0.004
KLK10	4	1.79 (1.00–3.20)	0.05	68.9%	0.022
KLK11	2	1.55 (0.64–3.76)	0.328	66.5%	0.084
KLK13	1	1.29 (0.98–1.71)	–	–	–
KLK14	2	1.43 (1.11–1.84)	0.041	16.1%	0.275
KLK15	1	1.12 (0.85–1.46)	–	–	–
Detection method					
qRT-PCR	9	2.12 (1.66–2.73)	< 0.001	0%	0.725
IHC	4	1.89 (1.27–2.81)	0.002	48.9%	0.118
ELISA	9	1.13 (1.06–2.81)	< 0.001	0%	0.725

*qRT-PCR* quantitative real-time polymerase chain reaction, *IHC* immunohistochemistry, *ELISA* enzyme-linked immunosorbent assay

Meta-regression analysis was also performed to explore the potential factors of the heterogeneity. We considered 4 covariates (ethnicity, sample size, KLKs member and detection method) may contribute to the heterogeneity. The results revealed that neither sample size, nor KLKs member was the source of heterogeneity, but the ethnicity populations and detection methods have influence on the pooled results (P < 0.05).

### Impact of KLKs expression on OS

For studies evaluating OS, moderate heterogeneity across studies was also observed (I^2^ = 51.9%, P = 0.001). Therefore, we also calculated the combined HR and the corresponding 95% CI based on a random model. According to the final pooled results, a significant correlation between KLKs overexpression and a worse OS was shown in patients with CRC with the pooled HR of 1.43 (95% CI 1.27–1.60, P < 0.001).

Because a substantial heterogeneity existed in the studies assessing OS, subgroup analysis was carried out (Table 4). It was revealed that elevated KLKs manifested itself as more indicative of shortened OS in Asian CRC patients (HR = 1.69, 95% CI 1.25–2.30, P < 0.001) than Caucasian cohorts (HR = 1.38, 95% CI 1.21–1.56, P < 0.001). In the subgroup stratified by KLKs member, four members of KLKs were evaluated by three or more studies and high levels of three members were associated with worse OS (KLK6: pooled HR = 1.47, 95% CI 1.16–1.86, P = 0.001; KLK7: pooled HR = 1.98, 95% CI 1.46–2.68, P < 0.001; KLK10: pooled HR = 1.90, 95% CI 1.01–3.58, P < 0.046) while that combined HR of KLK11 was 1.36 (95% CI 0.86–2.14) with P-value = 0.187. Importantly, the pooled results showed that elevated KLKs expression was significantly associated with worse OS by three method, with the combined HR being 2.06 (95% CI 1.57–2.72, P < 0.001) by qRT-PCR, 1.74 (95% CI 1.28–2.35, P < 0.001) by IHC and 1.18 (95% CI 1.09–1.28, P < 0.001) by ELISA.

**Table 4:** Results of subgroup analysis of studies for evaluating OS

Subgroup	Number of studies	HR (95% CI)	P_HR_	Heterogeneity (I^2^)	P_heterogeneity_
Ethnicity					
Caucasian	20	1.38 (1.21–1.56)	< 0.001	51.5%	0.004
Asian	5	1.69 (1.25–2.30)	< 0.001	47.2%	0.108
KLK member					
KLK4	1	1.07 (0.44–2.57)	–	–	–
KLK5	1	1.24 (1.05–1.47)	–	–	–
KLK6	6	1.47 (1.16–1.86)	0.001	53.6%	0.056
KLK7	4	1.98 (1.46–2.68)	< 0.001	6%	0.363
KLK8	2	1.36 (0.68–2.73)	0.380	76.8%	0.038
KLK10	4	1.90 (1.01–3.58)	0.046	69%	0.022
KLK11	3	1.36 (0.86–2.14)	0.187	47.4%	0.149
KLK13	1	1.36 (1.00–1.87)	–	–	–
KLK14	2	1.68 (1.12–2.51)	0.011	49.2%	0.161
KLK15	1	1.02 (0.77–1.36)	–	–	–
Detection method					
qRT-PCR	11	2.06 (1.57–2.72)	< 0.001	44%	0.058
IHC	5	1.74 (1.28–2.35)	< 0.001	30.6%	0.218
ELISA	9	1.18 (1.09–1.28)	< 0.001	0%	0.476

*qRT-PCR* quantitative real-time polymerase chain reaction, *IHC* immunohistochemistry, *ELISA* enzyme-linked immunosorbent assay

Then, a meta-regression analysis was carried out for the sources of the heterogeneity. It was indicated from the results that ethnicity and detection methods may have contributed to the heterogeneity.

### Publication bias and sensitivity analysis

**Fig. 3:**
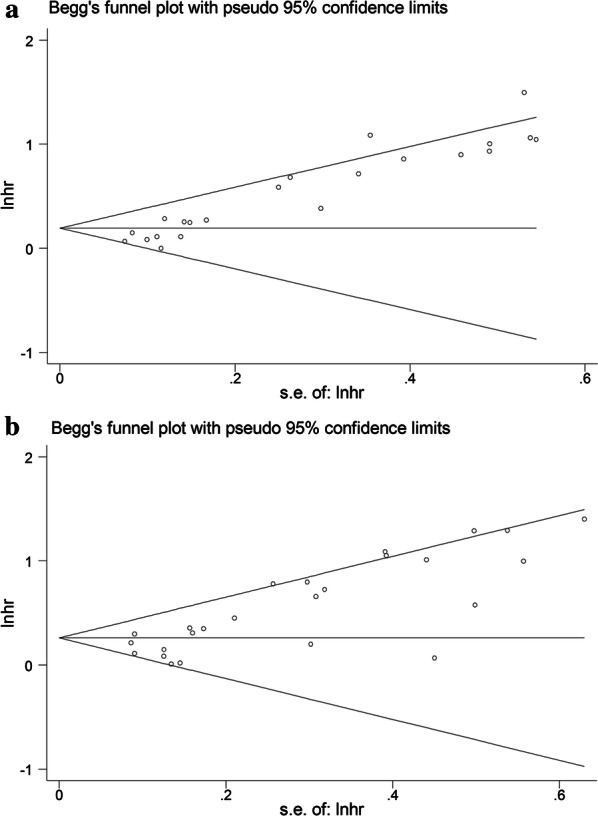
Begg’s funnel plots for the assessment of publication bias in the meta-analysis. **a** Funnel plot of the studies for DFS. **b** Funnel plot of the studies for OS

**Fig. 4:**
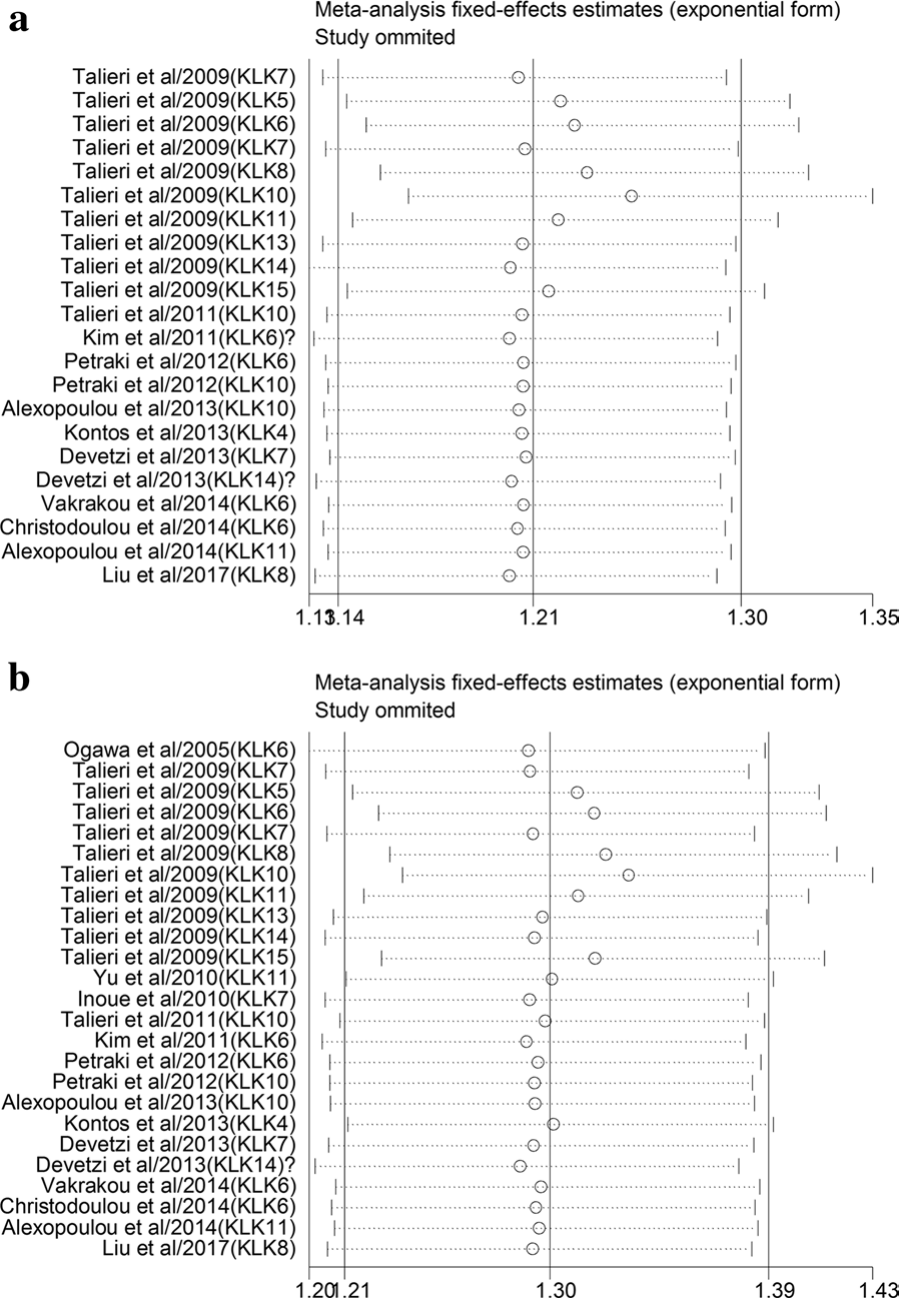
Sensitivity analysis of the meta-analysis. **a** Sensitivity analysis for DFS; **b** sensitivity analysis for OS

Begg’s funnel plot and Egger’s test were utilized to assess the presence of publication bias among the included literatures (Fig. 3). The funnel plots pointed out symmetry for all enrolled studies and Deeks’ test revealed potential heterogeneity in the present analysis about DFS and OS (P < 0.001). Then, we applied the trim and fill method to further investigate the publication bias and make the pooled HR more reliable both with the P value less than 0.01. Sensitivity analyses were further carried out to evaluate the stability of the pooled results for DFS and OS (Fig. 4). However, no specific study dominated the evidence synthesis as the removal of any individual study had no significant influence on the pooled results of DFS and OS, which indicates that the conclusions from our meta-analysis were relatively reliable.

## Discussion

Accumulating evidence has indicated that elevated KLKs promote cancer progression and predict poor prognosis of CRC patients. A series of quantitative analyses were conducted to investigate the prognostic value of KLKs overexpression in CRC. However, the sample sizes in most studies are small. Besides, it is inconclusive about the association between KLKs expression and progression of CRC. Thus, we performed this comprehensive and up-to-date research to draw a complete overview of all reported clinical studies investigating the impact of KLKs expression on prognosis of CRC patients.

To the best of our knowledge, the current study was the first systematic evaluation of the literatures to investigate the prognostic role of KLKs expression in CRC patients. We evaluated survival data from 25 different studies assessing OS and from 22 different studies estimating DFS. Our results suggest that the elevated level of KLKs is indeed a poor prognostic biomarker for CRC in OS (pooled HR = 1.43, 95% CI 1.27–1.60, P < 0.001) and DFS (pooled HR = 1.35, 95% CI 1.21–1.51, P < 0.001). In particular, the predictive roles for DFS and OS were more significant in Asians than in Caucasians. Additionally, when data was stratified according to detection methods, the results showed that the prognostic value of KLKs over-expression was significant in mRNA level and the protein level detected by qRT-PCR, IHC and ELISA, respectively. What’s more, several members of KLKs evaluated by more studies showed great promise for survival prediction including KLK6, KLK7 and KLK10. Of course, they along with the other members of KLKs worth further study. Moderate heterogeneity remained in the meta-analyses of the data for DFS and OS, which can be explained by ethnicity and detection methods in the meta-regression. Sensitivity analysis failed to identify any deviated study, indicating that there was high robustness in our meta-analysis.

Recently, accumulating researches have indicated KLKs to be potential predictors for CRC prognosis. Our data also revealed that KLKs are very promising for survival outcome prediction. However, several factors should be considered to be the necessary future directions of the application of KLKs in clinical practice. Firstly, an appropriate definition should be made about the standard cut-off value of KLKs level for increased survival risk. To a large extent, the methodological inconsistency contributed to the divergence of contemporary findings about the prognostic value of KLKs. Most researchers prefer median or mean value in their study as the approach to setting cut-off value of KLKs expression varied among different studies. Determination of standard patterns of KLKs expression will significantly prompt achievement of final consensus about the prognostic value of KLKs. Secondly, which should be used, the protein level or mRNA level? IHC, qRT-PCR and ELISA are all widely selected, detecting different levels of KLKs, respectively. Our results in this study also indicated that KLKs measured by these three methods all could be used to predict the survival outcome of CRC. Thirdly, which is better for clinical application, a single KLK or a panel of KLKs? CRC is a complex disease; single or limited biomarker is unlikely to reveal the complicated evolutionary process at the systemic level and lack of specificity and sensitivity. Our preliminary results show that combination biomarkers may be more reliable with greater power as they help to explain the internal mechanisms of CRC as well as the external factors influencing it [26, 27]. As a future perspective, combination of KLKs may be considered for the further improvement of the prognostic role and large-scale prospective studies are still necessary for further validation.

The associations between KLKs expression and cancer prognosis may be partly caused by the biological function of the KLKs. KLKs are secreted serine proteases with distinct expression patterns and physiological functions in several systems, especially in the digestive system. KLKs have been demonstrated to take part in numerous physiological processes such as cell growth regulation, angiogenesis, invasion, and metastasis. Aberrant expression of KLK family members is highly associated with various clinic-pathological parameters of patients suffered with colorectal, gastric, pancreatic, hepatic, and esophageal cancer. Accumulating evidence has revealed that KLKs facilitate CRC progression due to their ability to degrade extracellular matrix proteins, thereby promoting tumor invasion as well as metastasis [28]. A large number of studies have also demonstrated that KLKs play vital roles in regulating proteinase-activated receptors (PARs), which is a protein family containing four G-protein-coupled receptor members including PAR1, PAR2, PAR3, and PAR4 [29, 30]. Recent findings have indicated that KLKs has been inextricably linked to the cleavage of PARs, which may bring about coupling of the receptors to heterotrimeric G proteins, thereby generate signal transduction and thus enhance tumor cell proliferation [31]. It is also important to note that KLKs may play a significant part in uncoupling of the receptor from the signal transduction pathway with a strong implication on the initiation and progression of CRC as they are able to separate downstream of the PAR activation site or within an extracellular loop [32]. According to the previous evidence from studies on molecular mechanism, it is not difficult to understand why KLKs may become promising biomarkers for CRC prognosis prediction.

There are several important strengths from results of the current study. First, KLKs were confirmed to act as reliable prognostic biomarkers for CRC. Our data indicated that CRC patients with elevated expression levels of KLKs may suffer from an increased risk of poor survival, which was 1.43-fold higher for OS and 1.35-fold higher for DFS when compared with CRC patients with low KLKs expression. Second, we demonstrated that high KLKs expression correlated with poor OS and DFS both in Asian and Caucasian patients and this finding may be extended to other ethnic groups. Moreover, since some members of KLKs have been identified to have a biomarker role in the prognosis of CRC, future studies should be studied and uncovered the roles of other members of KLKs in CRC. In addition, as the easiest detection method, qRT-PCR may be recommended as the first choice since the prognostic value of KLKs by qRT-PCR was most significant in our study compared with ELISA and IHC. Finally, it underlines the potential to develop KLKs as a promising therapeutic target and prognostic biomarker for CRC. In the future, the KLKs may be accepted for clinical application like the common used tumor markers such as CEA, CA125, and CA199.

Apart from the inspiring outcomes, our conclusions should be interpreted with caution since there were also several limitations in the present work. To begin with, most of the included studies were designed as retrospective studies, which inherently contained greater potential for confounding than do randomised controlled trials. Next, TNM stage of the enrolled studies is a potent selection bias factor. Without patient-level data, it would be very difficult to tell whether KLKs are actually useful as a prognostic factor. A patient-level meta-analysis can be used to analyze all of the data in a consistent manner and includes data from unpublished studies. Moreover, only Asians and Caucasians were in the meta-analysis, no African population included in the analysis, which may cause potential heterogeneity from ethnicity. In addition, the numbers of studied were inconsistent among the KLKs including KLK1 (n = 5), KLK2 (n = 5), KLK4 (n = 1) and KLK5 (n = 1). Accordingly, subgroup analysis by specific member of KLKs could not be performed for the limited individual sample size. We ignored the variety of KLKs in the study. The meta-analysis was performed with studies of the selected KLKs, regardless of their types. Furthermore, the potential publication bias may exist in our analysis. At last, the current study is not registered and there may be a small offset, but we still strictly follow the steps of the systematic review.

## Conclusion

Taken together, in this study, it is concluded that tissue KLKs may be effectively predictive biomarkers for CRC prognosis. In future, more clinical studies are warranted to confirm the prognostic role of KLKs before its practical implementation in management of CRC.

## Data Availability

The data supporting the conclusions of this article is within the article.

## Abbreviations

CRC: Colorectal cancer
HR: Hazard ratio
CI: Confidence interval
PRISMA: Preferred Reporting Items for Systematic Reviews and Meta-analyses
NOS: Newcastle–Ottawa Scale
